# Game Transfer Phenomena and Problematic Interactive Media Use: Dispositional and Media Habit Factors

**DOI:** 10.3389/fpsyg.2021.585547

**Published:** 2021-04-22

**Authors:** Angelica B. Ortiz de Gortari, Jayne Gackenbach

**Affiliations:** ^1^Centre for the Science of Learning & Technology, University of Bergen, Bergen, Norway; ^2^Department of Psychology, MacEwan University, Edmonton, AB, Canada

**Keywords:** Game Transfer Phenomena, problematic video game playing, problematic social media use, gaming disorder, personality traits, fantasy proneness, gaming habits, game engagement

## Abstract

The study of the effects of interactive media has mainly focused on dysregulated behaviors, the conceptualization of which is supported by the paradigms of addiction. Research into Game Transfer Phenomena (GTP) examines the interplay between video game features, events while playing, and the manipulation of hardware, which can lead to sensory-perceptual and cognitive intrusions (e.g., hallucinations and recurrent thoughts) and self-agency transient changes (e.g., automatic behaviors) related to video games. GTP can influence the interpretation of stimuli and everyday interactions and, in contrast to gaming disorder, are relatively common and not necessarily negative. However, some players have reported feeling distress due to their GTP. This study focuses on how dispositional and interactive media habit factors are related to GTP and two forms of problematic interactive media [problematic video game playing (PVG) and problematic social media use (PSMU)]. A sample of 343 university students who played video games completed an online survey (58.7% male, 19–25 years old). Not all who had experienced GTP were identified as exhibiting PVG or PSMU, but all of those in the PVG group had experienced GTP. Overall, the profiles of the groups, including GTP (91.4%), PVG (28.5%), and PSMU (24.8%), were in accordance with previous findings. Those in the GTP and the PVG groups were characterized by being male, being highly engaged in the game (either while playing or *via* game-related activities), and showed preferences for game-related activities. However, while those in the GTP group were significantly more likely to be fantasy-prone, those with PVG were the ones who played most per day. Those in the PSMU group were characterized by being female and/or extroverted, frequently using social/sharing platforms, and seldom playing video games. A hierarchical binary logistic regression revealed that males were more likely to experience GTP. Increases in PVG, fantasy proneness, and neuroticism increased the odds of GTP. Future work can benefit from considering the role of GTP in gaming disorder, since intrusive thoughts, cognitive biases, and poor impulse control are pivotal in the initiation and maintenance of dysfunctional playing behaviors.

## Introduction

Playing video games and using social media can provide benefits such as informal learning, enabling creativity and self-expression, and belonging (Collin et al., 2011; Hall et al., 2012; Griffiths et al., 2017); on the other hand, they also tend to be associated with problematic use (Andreassen, 2015; Buono et al., 2020). The unhealthy and pathological use of interactive media has mainly been investigated with a focus on dysregulated use as supported by paradigms of addiction (Kuss and Griffiths, 2012; Billieux et al., 2019; Montag et al., 2019). The emerging area of research into Game Transfer Phenomena (GTP) examines the interplay between video game features, in-game experiences, and the manipulation of hardware, which can lead to sensory-perceptual and cognitive intrusions (e.g., hallucinations and recurrent thoughts) and self-agency transient changes (e.g., automatic behaviors) in relation to video games (Ortiz de Gortari, 2019b). It is only recently that GTP has started to be contextualized into research on gaming disorder to explain thought intrusions related to game content, reactivity to game-related cues, dissociation, and players’ distinctive relationships with their avatars (Ahn et al., 2015; Cudo et al., 2020; Stavropoulos et al., 2020). Gaming disorder (GD) only affects a small minority of individuals (Kardefelt-Winther et al., 2017; Przybylski et al., 2017). The worldwide prevalence of GD are 3.05% (Stevens et al., 2020) and can lead to clinically significant impairment or distress (Kuss and Griffiths, 2012; Montag et al., 2019). GTP is, in contrast to GD, relatively common and does not necessarily involve negative consequences, although distress and risks have been associated with GTP (Ortiz de Gortari et al., 2011, 2016; Ortiz de Gortari, 2019b). The prevalence rate of GTP (having experienced at least one instance of GTP) is estimated to range between 82 and 96% in studies conducted with international samples (*N* > 6,000, 15–60 years old) (Ortiz de Gortari, 2017; Dindar and Ortiz de Gortari, 2017; Ortiz de Gortari and Griffiths, 2016b).

This paper examined and compared GTP with two forms of problematic media use [i.e., problematic video game playing (PVG) and problematic social media use (PSMU)].

## Literature Review

### Game Transfer Phenomena

Game transfer phenomena is in the continuum between everyday involuntary phenomena (e.g., auditory or visual imagery) to unusual phenomena that are commonly linked with psychopathology (e.g., hallucinations, obsessive thoughts, dissociations) (Ortiz de Gortari, 2019a). Participants in studies on GTP have consisted of non-clinical samples (i.e., no clinical diagnosis or drug use) from the general population who have not been identified as having a gaming disorder. The existing studies have not been attached to specific video game genres, although research has been conducted on specific mobile augmented reality games (Sifonis, 2018; Ortiz de Gortari, 2019a).

Game transfer phenomena has mainly been reported in awake states after playing, but it has also been experienced when trying to fall asleep (Ortiz de Gortari and Griffiths, 2016b). Experiments have shown that playing video games can lead to replays of the game manifesting as images or sounds during the onset of sleep (Wamsley et al., 2010; Kusse et al., 2012). Interestingly, hypnagogic images have even been reported by amnesic patients, suggesting that implicit memory (information acquired unconsciously) rather than explicit memory (conscious and intentional recollection) mediates these forms of GTP (Stickgold et al., 2000).

Game transfer phenomena has mainly been reported *after* playing, but in some circumstances and for certain games, players have reported experiencing GTP *while* playing (Ortiz de Gortari and Griffiths, 2016b). This is particularly true for augmented reality games that require switching between the virtual and the real world (Sifonis, 2018; Ortiz de Gortari, 2019a). However, GTP should not be confused with subjective phenomena that occur while playing, such as immersion, flow, and subjective presence in the virtual world, or losing track of time that some have conceptualized as game engagement (Brockmyer et al., 2009).

In most cases, GTP is not associated with negative consequences and tends to be appraised by players as pleasurable experiences (Ortiz de Gortari and Griffiths, 2016b). Positive and even therapeutic uses of GTP have been proposed (Ortiz de Gortari, 2018), including the potential to induce GTP to interfere with distressful thoughts or images due to traumatic events (Ortiz de Gortari and Griffiths, 2016a). However, a large number of those who have experienced GTP in several forms frequently reported distress and dysfunction (Ortiz de Gortari et al., 2016). GTP has also been reported to provoke confusion and sleep deprivation, and it has been associated with risk-taking behaviors (Ortiz de Gortari and Griffiths, 2013, 2014). It is not clearly understood when GTP can be hazardous. According to Ortiz de Gortari (2019b), the potential for GTP to become problematic depends on a number of different factors: (i) the individual’s interpretation of their GTP experience (e.g., recognizing GTP as a consequence of playing rather than casting doubts about mental stability); (ii) the frequency and duration (e.g., many times per day, once per week, or for hours or seconds); (iii) the perceived location of the images and sounds, which can be either inner or outer phenomena (e.g., hearing sounds in the head as opposed to hearing sounds coming from somewhere else); (iv) the circumstances where GTP occurred (e.g., in compromised situations such as driving or while lying in bed); (vi) how the individual acts when experiencing GTP (e.g., getting distracted from the task at hand, acting out toward game-related stimuli, which, under certain circumstances such as driving, can be dangerous); and (vi) the content of the experience (e.g., seeing abstract video game shapes or realistic video game content or hearing an aversive or soothing sound).

### Problematic Media Use

Problematic online behaviors and activities are usually examined from two different approaches (Davis, 2001; Montag et al., 2015). Problematic use of media not concerning any specific online activity is referred to as Generalized Problematic Internet Use or Generalized Problematic Media Use. The second approach focuses on specific online behaviors (Laconi et al., 2015), such as online gambling, cybersex, and shopping, as well as playing online video games or using social networking sites that are examined in the current study.

Gambling addiction is the only non-substance addictive behavior recognized in the fifth revision of the Diagnostic and Statistical Manual of Mental Disorders (DSM-5) (American Psychiatric Association, 2013). “Internet Gaming Disorder” is included as a condition that requires further study. More recently, PVG, referred to as Gaming Disorder, received recognition by the World Health Organization (WHO) (2018) in the 11th Revision of the International Classification of Diseases (ICD-11). Therefore, caution is warranted to distinguish actual Gaming Disorder, which is characterized by a sum of symptoms endured for a sustained period of time that evolve into dysfunction from high engagement (e.g., frequent playing without presenting a problematic outcome), and to distinguish Gaming Disorder from transient or episodic periods of intense playing that can become problematic (Kardefelt-Winther et al., 2017; Billieux et al., 2019; Männikkö et al., 2020).

In terms of characteristics, playing video games and using social media differ substantially, even though some social media sites include video games, and many video games have a social component (e.g., Xbox live). For instance, playing video games requires focusing on earning rewards, mastering challenges, visual–motor coordination, involvement in fantasy, detachment from reality, and sometimes socializing (Ortiz de Gortari and Griffiths, 2017). In contrast, social media use involves communication; sharing thoughts, photos, and videos; consuming information; and sometimes playing social games.

The preference for different kinds of media and activities according to different individual profiles is thus expected. Differences have been established in terms of dispositional characteristics between video game players and avid social network users. Video game players tend to be males, although in recent years, more females have started to play video games (Lopez-Fernandez et al., 2019). Males usually have more problems with their video game playing, while females are usually more active social media users.

While video game players are usually considered tech-savvy, introverted, interested in sensation seeking, desiring to escape from reality, and fantasy-prone (Mehroof and Griffiths, 2010; Ballabio et al., 2017), social media users tend to be apprehensive about real life, be more involved in others’ lives, and compare their own lives with others (Lopez-Fernandez, 2018).

Problematic playing and gaming disorder have been connected with a variety of personality traits, including neuroticism, high impulsivity, and high aggressiveness (Gervasi et al., 2017) while also being negatively associated with extroversion, openness to experience, agreeableness, and conscientiousness (Montag et al., 2011). PSMU has been shown to be positively correlated with neuroticism and extroversion (Andreassen et al., 2012).

### Aim and Study Justification

To date, little is known about the dispositional factors involved in experiencing GTP and their relationship with the problematic use of other interactive media. Therefore, for the first time, this study explores the interrelationship and areas of overlap between GTP and two forms of problematic media use, herein referred to as PVG and PSMU. This study mainly focuses on examining those personality traits that reflect attitudes, behaviors, feelings, and thoughts that have been found to increase one’s vulnerability to problematic media use (Montag et al., 2011; Collins et al., 2012). A further objective is the examination of the habits that are intrinsic to the use of interactive media, such as consumption patterns (e.g., time spent playing and frequency of playing), engagement during playing, and preferences for game-related activities. The following specific research questions are addressed:

(1) Is GTP associated with PVG and PSMU, and what is their prevalence in the sample? Evidence suggests that there is a relationship between GTP and PVG (Ortiz de Gortari et al., 2016), but the relationship between PSMU and GTP has not previously been investigated.
(2) Which video game and social media habits can be observed among those who have experienced GTP and have PVG or PSMU? Spending excessive time playing video games and using social media are core characteristics of PVG and PSMU, respectively. However, excessive playing time appears not to be needed to experience GTP (Ortiz de Gortari, 2019a).
(3) Does engagement in a game differ for those who have experienced GTP as opposed to those who have PVG or PSMU? Game engagement has been conceptualized in different ways. Some consider it to be the subjective experience that involves flow, absorption, immersion, and presence (Brockmyer et al., 2009); this is best understood as engagement while playing. Others understand game engagement as a person’s commitment to his or her gaming activities beyond the gameplay itself (e.g., reading about video games, talking with friends about games, or preferring playing over other activities) (Becerra, 2012). To be engaged in a game also defines those players who only exhibit the primary peripheral criteria of a gaming disorder, such as salience, tolerance, and mood modification rather than conflict, withdrawal, relapse, and other problems due to playing (Charlton and Danforth, 2007; Krossbakken et al., 2018).
(4) Is gender associated with GTP, PVG, or PSMU, and if so, which gender is predominant among those who have experienced GTP compared to those who have PVG or PSMU? Most studies have found no association between gender and GTP (Ortiz de Gortari and Griffiths, 2016b; Dindar and Ortiz de Gortari, 2017; Ortiz de Gortari, 2017). However, one study conducted on a mobile phone augmented reality game showed that females were more likely to experience GTP (Sifonis, 2018). Furthermore, males have been found to be more vulnerable to PVG (e.g., Kuss and Griffiths, 2012), while females are more likely to be at risk of PSMU (e.g., Andreassen et al., 2016).
(5) Which personality traits characterize those who have experienced GTP or have PVG or PSMU? Certain personality traits, such as openness to experiences and fantasy proneness, have been suggested to be relevant for GTP (Ortiz de Gortari et al., 2016). Problematic playing has been associated with different personality traits than PSMU (Montag et al., 2011; Andreassen et al., 2012; Gervasi et al., 2017).

Problematic media use is examined in this paper considering patterns of behaviors that involve conflict and dysregulated use of media, but which do not necessarily connote a severity of clinical relevance. The findings should be interpreted as pointing to *a risk* of developing PVG and PSMU; thus, this study avoids erroneous inflation of problems associated with interactive technologies.

## Materials and Methods

### Participants

A total of 481 participants completed an online survey on playing video games and social media use. Participants who did not play video games were excluded. The final sample consisted of 343 respondents. Of these, 58.7% were male and 41.3% were female. Most participants were 19 years old or younger (49%), with the next largest group being those 20–25 years old (46.6%). Most stated that they were single (73%). All participants were students at a western Canadian university who received course credit for their participation.

### Procedure

After informed consent was obtained, which was developed in accordance with the requirements of the ethics committee of the university, respondents were asked to provide three demographic pieces of information: gender, age, and marital status. Questions and scales about media consumption, video game engagement, individual characteristics, and dream-related information were included in the main part of the survey. The online survey was available for student participation over the entire academic year 2017–2018. The measures used to answer the research questions of the study are described below.

### Materials

The Game Transfer Phenomena Scale (GTPS) is a 20-item Likert-type scale of frequency with responses ranging from 1 (“never”) to 5 (“all the time”) (Ortiz de Gortari et al., 2015). The subscales included Altered Perceptions Modality, which is subdivided into visual, auditory, and body sensory perceptions (e.g., “I have visualized or seen video game images with closed eyes” or “I have heard sounds, music, or voices from the game”); the Automatic Mental Processes Modality (e.g., “I have thought about using something from a video game in real life”); and the Actions and Behavior Modality (e.g., “I have sung, shouted, or said something from a video game in real life without intending to do so”). Based on previous studies (Ortiz de Gortari and Griffiths, 2016b; Dindar and Ortiz de Gortari, 2017), a composite score was calculated as the sum of the GTP items. Reporting at least one GTP item was considered having experienced GTP. The overall Cronbach’s alpha for the 20-item scale in the present study was 0.938, and the McDonald’s omega was 0.939.

The Problem Video Game Playing Questionnaire (PVGQ) is a nine-item scale scored with “yes” or “no” responses that was developed to assess PVG (Tejeiro Salguero and Morán, 2002) based on criteria borrowed from pathological gambling and substance dependence in the DSM-IV (APA, 2000). This was one of the first validated scales to measure PVG that was initially developed for adolescents but then used with the general adult population. The items measure various components of addiction, such as preoccupation, tolerance, loss of control, withdrawal, escape, lies and deception, disregard of physical or psychological consequences, and family/school/work disruption. Certain items are explicitly related to conflicts due to playing, such as the following: “In order to play video games, I have skipped classes or work, lied, stolen, or had an argument or a fight with someone.” Other items assess what has been considered as losing control: “I have tried to control, cut back or stop playing, or I usually play with the video games over a longer period than I intended” or “I spend an increasing amount of time playing video games.” Tejeiro Salguero et al. (2012) recommended a cutoff point of 4 to be considered having PVG. However, some authors have argued a cutoff point of 4 to be inadequate. Hart et al. (2009) did not find evidence of problematic playing when using 4 as cutoff point. Moreover, some authors have argued that half or more of the diagnostic criteria should be met to be classified with problematic playing (Lemmens et al., 2009). In this study, a cutoff point of 5 was used to determine PVG, as suggested by Lopez-Fernandez et al. (2014). The Cronbach’s alpha of this measure for this study was 0.713; the McDonald’s omega was 0.721.

The Questionnaire on Video Game Consumption Habits (VGCH) consists of a 24-item questionnaire to measure video game consumption habits and general preferences for video games (Becerra, 2012), including degrees of attraction to video games, concerns about video games, and academic and non-academic interferences. The questionnaire includes single categorical items about video game consumption such as the experience of playing video games, the frequency of playing, and daily playing time. The questions about the number of video games played and name of the favorite game were excluded in the current study. The questionnaire also includes 19 items measured with a 5-point Likert-type scale of agreement. Examples of the items included “Video games seem fun,” “I go to bed late, and I get up early to keep playing,” “I save money to spend on video games,” or “I talk to my friends about video games.” Other items determine a participant’s preferences for playing over other activities, with some items overlapping with those in the PVG measure to a certain degree, such as the following: “I dedicate more time to video games than to doing homework” and “I dedicate more time to video games than to being with my family.” Moreover, some items measure flow and immersion (e.g., “When I play video games, time flies”). Cronbach’s alpha of the 19 items for this study was 0.954; the McDonald’s omega was 0.933.

The Bergen Social Media Addiction Scale (BSMAS) takes portions of a previous scale, the Bergen Facebook Addiction Scale (BFAS) (Andreassen et al., 2016), as, over the years, social networking has had a drastic increase in different media. No longer is social networking equated with Facebook, since there are now other popular media platforms, including Twitter, Instagram, and Pinterest. The six-item scale reflects six “addiction elements” (salience, mood modification, tolerance, withdrawal, conflict, and relapse) reported by the participant regarding social media usage in the previous year. Participants answer on a 5-point scale, with responses ranging from 1 (“very rarely”) to 5 (“very often”). The items include questions such as the following: “How often during the last year have you used social media to forget about personal problems?” A score over 19 was considered the cutoff point for being classified with PSMU based on Bányai et al. (2017). The Cronbach’s alpha for this study was 0.811; the McDonald’s omega was 0.815.

Additionally, a total of nine different types of applications were included in the questionnaire, although only eight applications received enough participant data to be part of the analyses of frequent use of social media and sharing platforms such as Facebook, YouTube, and Instagram (MUF) (Gackenbach et al., 2016).

The Game Engagement Questionnaire (GEQ) is a 19-item scale (Brockmyer et al., 2009) that was developed to indicate how the participant perceives the reality around them while they are playing video games. The scale can be used with any video game genre, but it focuses explicitly on measuring absorption in the video game and dissociation from reality. Responses are provided in the form of “yes” or “no” answers to questions like “I lose track of time [while playing video games]” or “I don’t answer when someone talks to me [while playing video games].” Total scores were computed by summing up the response scores. The Cronbach’s alpha for this study was registered at 0.855; the McDonald’s omega was 0.857.

The Big Five Inventory (BFI) is a scale with 44 items that are used to determine which traits a participant has that ultimately determine the extent the Big Five Factors play a part in their personality (John and Srivastava, 1999). On a scale from 1 (“strongly disagree”) to 5 (“strongly agree”), participants are to respond to various statements about what they can observe about themselves in certain situations. The following are examples of these statements: “I am someone who does a thorough job” or “I am someone who starts quarrels with others.” Total scores were computed by summing up the response scores per personality trait. Cronbach’s alpha for this study was 0.702; the McDonald’s omega was 0.703.

The Creative Experiences Questionnaire (CEQ) was developed as a 25-item questionnaire scored with “yes” or “no” responses to determine fantasy proneness (Merckelbach et al., 2001). Sample questions include the following: “Many of my fantasies have a realistic intensity” and “When I think of something cold, I actually get cold.” The researcher can determine the participant’s fantasy proneness based on his or her responses. Some items of the questionnaire deal with “intense elaboration of and profound involvement in fantasy and daydreaming,” while others address the “consequences of fantasizing.” A composite score was computed based on the sum of the items. Cronbach’s alpha for this study was 0.720; the McDonald’s omega was 0.712.

### Statistical Analysis

The statistical analysis was performed using the SPSS package (SPSS 26 for Windows; SPSS Inc., Chicago, IL, United States). Bivariate and multivariate statistics using non-parametric and parametric tests were performed. Multiple imputations were computed to analyze the patterns of the missing data. The missing data were completely random and below 2%. The missing cases were not imputed since a rate of 5% or less had been found to be inconsequential, and statistical analyses are considered to be biased with 10% missing data (Dong and Peng, 2013). Preliminary analyses ensured that there was no violation of the assumptions in each of the tests performed. In order to compare each pair of the groups, including GTP/No GTP, PVG/No PVG, and PSMU/No PSMU, dichotomous variables were created for GTP based on having experienced GTP at least once (Ortiz de Gortari and Griffiths, 2016b) and at the recommended cutoff points for classifying participants as having PVG and PSMU, respectively (Tejeiro Salguero et al., 2012; Bányai et al., 2017). Spearman rank-order correlations and Mann–Whitney *U* tests were used with non-normal distributed continuous variables, and chi-square (χ^2^) tests were used for categorical variables. The last step consisted of performing a hierarchical binary logistic regression for GTP. All statistics were two-tailed with a 0.05 level of significance, and the effect sizes were computed. A Bonferroni correction was applied to obtain an adjusted *p*-value. The original alpha level (0.05) was divided by the number of tests performed in each *t*-test, and the categories in the chi-square test. Bonferroni correction has been recommended when a large number of tests are conducted without preplanned hypotheses previously collected for the data and for protection against Type I error (Armstrong, 2014).

## Results

### Prevalence and Interplay Between Game Transfer Phenomena, Problematic Video Game Playing, and Problematic Social Media Use

Most participants had experienced at least one instance of GTP (91.3%, *n* = 298); 86% had experienced more than one type of GTP, and 71.1% had experienced five or more different types of GTP (based on the 20 items of the GTP). The types of GTP reported by at least half of the sample were hearing music from a video game (69.4%), seeing or visualizing images (74.3%), thinking about using something from a video game in real life (61.4%), misinterpreting sounds (59.7%), wanting or feeling the urge to do something in real life after seeing something that reminds one of the video game (55.6%), and hearing sounds from a video game (55.1%). The least common type of GTP was mixing up video game events with actual real-life events (21.4%).

Correlation coefficients showed only a strong positive correlation between GTP and PVG [*r*s(318) = 0.51, *p* < 0.001]. No correlation was found between GTP and PSMU or between PVG and PSMU. Almost one third were classified with PVG (28.5%, *n* = 95) and one in four with PSMU (24.8%, *n* = 136) (see the “Measures” section for the cutoff points used). Moreover, the chi-square tests showed that 31.0% of those who had experienced GTP had PVG, and 24.1% had PSMU. A significant association was only found between GTP and PVG [χ^2^(1) = 12.120, *p* < 0.001], although the effect size was small. Those who did not have PVG were significantly less likely to have experienced GTP. Every participant considered as having PVG had also experienced GTP.

### Demographics

The chi-square tests showed significant differences in terms of gender for GTP, PVG, and PSMU, but not in terms of age. There were more males in the GTP group, and females were significantly more likely to not have experienced GTP [χ^2^(1) = 7.792, *p* < 0.01]. There were also more males in the PVG group, and in this case, males were significantly more likely to be in the PVG group, while females were significantly less likely to be in the PVG group [χ^2^(1) = 19.062, *p* < 0.001]. There were more females in the PSMU group, and females were significantly more likely to be in the PSMU group, while males were significantly less likely to be in the PSMU group [χ^2^(1) = 5.119, *p* < 0.05]. Overall, the effect sizes were small (see Table 1 for the full results).

**TABLE 1:** Chi-square of GTP, PVG, and PSMU on demographics.

	GTP %	No GTP %	χ^2^	*df*	Effect size ^b^	PVG %	No PVG %	χ^2^	*df*	Effect size ^b^	PSMU %	No PSMU %	χ^2^	*df*	Effect size ^b^
*Gender*	*n* = 296	*n* = 28	7.792**	1	0.16	*n* = 94	*n* = 237	19.062***	1	0.11	*n* = 82	*n* = 251	5.119*	1	0.10
Male	59.5	32.1				77.7	51.5				47.6	61.8			
*SR*^a^	(0.5)	(−1.7)				(2.4)	(−1.5)				(−1.3)	(0.7)			
Female	40.5	67.9				22.3	48.5				52.4	38.2			
*SR*	(−0.6)	(2.0)				(−2.8)	(1.8)				(1.5)	(−0.9)			
*Age*	*n* = 298	*n* = 28	2.560	2		*n* = 95	*n* = 238	3.701	2		*n* = 83	*n* = 252	3.025	2	
19 years or younger	50.3	35.7				48.4	50.0				53.0	47.6			
*SR*	(0.3)	(−1.0)				(−0.2)	(0.1)				(−0.5)	(−0.3)			
20–25 years	46.0	57.1				50.5	44.5				45.8	46.8			
*SR*	(−0.2)	(0.8)				(0.6)	(−0.4)				(−0.1)	(0.1)			
26 years or older	3.7	7.2				1.1	5.5				1.2	5.6			
*SR*	(−0.3)	(0.8)				(−1.5)	(0.9)				(−1.4)	(0.8)			

*p < 0.05, **p < 0.01, ***p < 0.001.

*^*a*^Standardized Residual.*

*^*b*^Effect size = Cramer’s V. GTP, Game Transfer Phenomena; PSMU, problematic social media use; PVG, problematic video game playing*.

### Personality Traits

According to the Spearman rank-order correlations, there were positive correlations between GTP, openness, and fantasy proneness as measured by the CEQ. Also, positive correlations were found between PVG and CEQ. Positive correlations were also found between PSMU and neuroticism, extroversion, and the CEQ scores (Table 2).

**TABLE 2:** Spearman correlation matrix.

		1	2	3	4	5	6	7	8	9	10	11	12
(1)	GTP	1.000	0.526**	0.060	0.525**	0.473**	0.383**	0.038	−0.090	−0.028	0.080	0.150**	−0.069
(2)	PVG		1.000	−0.038	0.547**	0.726**	0.137*	−0.082	−0.053	−0.076	0.099	−0.020	−0.080
(3)	PSMU			1.000	0.154**	−0.208**	0.192**	0.118*	0.067	−0.060	0.123*	−0.054	0.508**
(4)	GEQ				1.000	0.463**	0.349**	−0.045	−0.063	−0.037	0.187**	0.071	0.020
(5)	VGCH					1.000	0.086	−0.129*	−0.057	−0.034	0.089	0.060	−0.185**
(6)	CEQ						1.000	0.141*	0.025	−0.013	0.241**	0.377**	0.098
(7)	Extroversion							1.000	−0.002	0.212**	0.004	0.310**	0.085
(8)	Agreeableness								1.000	0.096	−0.070	0.118*	0.069
(9)	Conscientiousness									1.000	0.116*	0.177**	−0.080
(10)	Neuroticism										1.000	0.153**	0.066
(11)	Openness											1.000	−0.122*
(12)	MUF												1.000

**Correlation is significant at the 0.01 level (two-tailed).

*Correlation is significant at the 0.05 level (two-tailed).

*GTP, Game Transfer Phenomena; PSMU, problematic social media use; PVG, problematic video game playing; GEQ, Game Engagement Questionnaire; VGCH, Questionnaire on Video Game Consumption Habits; CEQ, Creative Experiences Questionnaire; MUF, Media Use Frequency.*

According to the Mann–Whitney *U* test, comparing the GTP with the No GTP group showed that CEQ scores were significantly higher in the GTP group. There were no significant differences when comparing the PVG with the No PVG group. Comparing the PSMU with the No PSMU group showed that extroversion scores were significantly higher in the PSMU group. All the effect sizes were small (see Table 3 for the full results).

**TABLE 3:** Mann–Whitney U test of GTP, PVG, and PSMU on personality traits.

Variables	Groups	*n*	*M*	*SD*	Median	Mean rank	*U*	*Z*	*p*	Effect size^a^
Extroversion	GTP	294	25.58	5.06	26.00	161.05	3,984.0	−0.281	0.779	
	No GTP	28	25.86	5.89	26.00	166.21				
	PVG	95	24.77	5.64	25.00	149.10	9,604.5	−1.828	0.068	
	No PVG	232	26.09	4.81	26.00	170.10				
	PSMU	80	27.05	4.75	27.00	190.94	7,884.5	−2.811	0.005*	0.16
	No PSMU	249	25.27	5.13	25.00	156.66				
Agreeableness	GTP	290	31.91	4.44	31.50	156.07	3,066.0	−1.581	0.114	
	No GTP	26	33.73	5.74	34.50	185.58				
	PVG	93	31.81	4.52	31.00	156.79	10,210.5	−0.580	0.562	
	No PVG	229	32.08	4.56	32.00	163.41				
	PSMU	79	32.78	4.26	32.00	179.65	8,323.0	−1.876	0.061	
	No PSMU	245	31.69	4.67	31.00	156.97				
Conscientious	GTP	294	31.00	4.23	31.00	157.90	3,059.0	−1.977	0.048	
	No GTP	27	32.81	5.21	33.00	194.70				
	PVG	95	30.49	4.61	31.00	153.60	10,032.0	−1.333	0.183	
	No PVG	233	31.38	4.28	32.00	168.94				
	PSMU	82	30.94	4.11	30.50	159.30	9,660.0	−0.680	0.496	
	No PSMU	248	31.12	4.51	31.00	167.55				
Neuroticism	GTP	293	25.86	4.91	26.00	164.92	2,953.0	−2.455	0.014	
	No GTP	28	23.43	4.77	24.00	119.96				
	PVG	95	26.22	4.85	26.00	172.79	10,374.5	−0.949	0.342	
	No PVG	234	25.58	4.90	26.00	161.84				
	PSMU	81	26.23	4.91	26.00	176.59	9,186.0	−1.207	0.227	
	No PSMU	249	25.44	4.97	26.00	161.89				
Openness	GTP	292	35.68	5.60	36.00	164.06	3,048.5	−2.226	0.026	
	No GTP	28	33.43	5.32	33.00	123.38				
	PVG	95	35.56	5.21	36.00	163.24	10,947.5	−0.094	0.925	
	No PVG	232	35.53	5.70	36.00	164.31				
	PSMU	81	35.57	5.07	36.00	166.84	9,895.0	−0.201	0.841	
	No PSMU	248	35.52	5.69	36.00	164.40				
CEQ	GTP	275	10.02	3.94	10.00	158.31	1,565.5	−4.751	0.000***	0.27
	No GTP	26	6.04	3.34	5.50	73.71				
	PVG	89	10.47	4.14	11.00	170.90	8,196.5	−2.138	0.032	
	No PVG	218	9.32	3.94	9.00	147.10				
	PSMU	75	10.37	4.33	10.00	168.96	7,578.0	−1.684	0.092	
	No PSMU	232	9.50	4.01	9.50	149.16				

*p < 0.05, **p < 0.01, ***p < 0.001.

*M, arithmetic; mean; SD, standard deviation; Me, median.*

*^*a*^Effect size = Cohen’s.*

*GTP, Game Transfer Phenomena; PSMU, problematic social media use; PVG, problematic video game playing; CEQ, Creative Experiences Questionnaire.*

### Media Consumption

The majority of the participants had been playing video games for more than 4 years (81.8%). Most played either every day (29.2%) or once a month (27.1%), either for less than 1 h per day (33.1%) or 1–2 h per day (28.4%). The favorite game titles listed among the participants were *League of Legends*, *Halo*, *Overwatch*, *Sims*, *Fortnite*, *Call of Duty*, *Mario Kart*, *NHL*, *Skyrim*, and *The Last of Us.* The most popular platforms used (i.e., checking once per hour or more often) by the full sample were Instagram (35.4%), YouTube (21.3%), and Facebook (16.8%).

#### Gaming Habits

Chi-square tests were conducted to examine experience playing, frequency of playing, and daily playing time. All the effect sizes were large (see Table 4 for the full results).

**TABLE 4:** Chi-square of GTP, PVG, and PSMU on media use habits.

	GTP %	No GTP %	χ^2^	*df*	Effect size^a^	PVG %	No PVG %	χ^2^	*df*	Effect size^a^	PSMU %	No PSMU %	χ^2^	*df*	Effect size^a^
*Daily playing time*	*n* = 265	*n* = 15	9.151^1^	3		*n* = 92	*n* = 195	63.329***	3	0.81	*n* = 63	*n* = 226	2.517	3	
Less than 1 h/day	30.9	73.3				5.4	45.6				39.7	31.9			
*SR*	(−0.6)	(2.7)				(−4.6)	(3.1)				(0.8)	(−0.4)			
1–2 h/day	29.9	13.3				26.1	29.2				30.2	27.8			
*SR*	(0.3)	(−1.1)				(−0.4)	(0.3)				(0.3)	(−0.1)			
2–3 h/day	19.2	6.7				31.5	14.9				17.5	20.8			
*SR*	(0.3)	(−1.1)				(2.4)	(−1.7)				(−0.5)	(0.2)			
More than 3 h/day	20.0	6.7				37.0	10.3				12.7	19.5			
*SR*	(0.3)	(−1.1)				(4.0)	(−2.8)				(−1.0)	(0.5)			
*Experience playing*	*n* = 296	*n* = 25	7.976^1^	3		*n* = 95	*n* = 231	17.551***^1^	3	0.43	*n* = 83	*n* = 245	4.966^1^	3	
Months	9.1	24.0				1.1	13.9				13.3	9.0			
*SR*	(−0.6)	(2.1)				(−2.8)	(1.8)				(0.9)	(−0.5)			
A year	0.7	4.0				0.0	1.3				1.2	0.4			
*SR*	(−0.5)	(1.6)				(−0.9)	(0.6)				(0.7)	(−0.4)			
2–3 years	8.1	4.0				5.2	8.2				10.8	6.1			
*SR*	(0.2)	(−0.7)				(−0.8)	(0.5)				(1.2)	(−0.7)			
More than 4 years	82.1	68.0				93.7	76.6				74.7	84.5			
*SR*	(0.2)	(−0.7)				(1.3)	(−0.8)				(−0.7)	(0.4)			
*Frequency of playing*	*n* = 298	*n* = 28	26.216***	3	0.49	*n* = 95	*n* = 238	93.037***	3	0.92	*n* = 83	*n* = 252	11.328*	3	0.32
Once a month	24.8	67.9				6.3	35.3				39.8	22.6			
*SR*	(−1.2)	(3.9)				(−3.9)	(2.5)				(2.3)	(−1.3)			
Weekends	21.5	21.4				4.2	28.2				21.7	22.1			
*SR*	(0.0)	(0.0)				(−3.6)	(2.3)				(−0.1)	(0.0)			
3–4 days per week	23.2	7.1				26.3	21.0				20.5	23.0			
*SR*	(0.5)	(−1.7)				(0.8)	(−0.5)				(−0.4)	(0.2)			
Every day	30.5	3.6				63.2	15.5				18.1	32.1			
*SR*	(0.8)	(−2.5)				(6.1)	(−3.9)				(−1.8)	(1.0)			

*p < 0.05, **p < 0.01, ***p < 0.001.

*^1^Fisher exact test was computed when more than 20% of expected counts were less than 5.*

*SR, standardized residual.*

*^*a*^Effect size = Cramer’s V.*

*GTP, Game Transfer Phenomena; PSMU, problematic social media use; PVG, problematic video game playing*.

##### Game transfer phenomena and playing habits

Comparing the GTP vs. the No GTP group showed no differences in terms of experience playing or daily playing time. Only significant differences were found for the frequency of playing. However, *post hoc* test showed that only playing once a month in comparison to playing more frequently (i.e., weekends, three to four times per week, every day) was significantly associated (SR = 3.9) with the No GTP group [χ^2^(3) = 26.216, *p* < 0.001].

##### Problematic video game playing and playing habits

Comparing the PVG vs. the No PVG group showed significant differences in terms of experience playing [χ^2^(3) = 17.551, *p* < 0.001], daily playing time [χ^2^(3) = 63.329, *p* < 0.001], and frequency of playing [χ^2^(3) = 93.037, *p* < 0.001]. According to *post hoc* tests, only those who had been playing video games for months were significantly less likely (SR = −2.8) to be in the PVG group. Moreover, according to the *post hoc* test performed with the frequency of playing, only playing every day was significantly more likely (SR = 6.1) to be associated with the PVG group. Lastly, *post hoc* test showed that playing 2–3 h a day (SR = 2.4) or playing more than 3 h a day (SR = 4.0) was significantly more likely to be associated with the PVG group.

##### Problematic social media use and playing habits

Comparing the PSMU vs. the No PSMU group in terms of playing habits showed only significant differences in frequency of playing [χ^2^(3) = 11.328, *p* < 0.001]. The *post hoc* test showed that only playing once a month (SR = 2.3) was significantly more likely to be associated with PSMU.

#### Video Game Engagement

The Spearman rank-order correlations showed that GTP and PVG were positively correlated with GE and VGCH scores. PSMU was positively correlated with GE scores and negatively correlated with VGCH (Table 2).

Further analyses conducted with the Mann–Whitney *U* test showed that when comparing the GTP vs. the No GTP group, engagement while playing (GE score) and engagement in game-related activities and preference for playing (VGCH score) were statistically significantly higher in the GTP group. Both effect sizes were of medium size. Comparing the PVG vs. the No PVG group showed that GE and VGCH scores were statistically significantly higher in the PVG group. The effect size for GE scores was medium, while it was large for VGCH scores. Comparing the PSMU vs. the No PSMU group showed that only the VGCH scores were statistically significantly lower in the PSMU group, and this effect size was small (see Table 5 for the full results).

**TABLE 5:** Mann–Whitney test of GTP, PVG, and PSMU on video game engagement and social media.

Variables	Groups	*n*	*M*	*SD*	Me	Mean rank	*U*	*Z*	*p*	Effect size^a^
GEQ^1^	GTP	281	37.56	6.93	38.00	161.99	1,128.0	−5.630	0.000***	−0.33
	No GTP	25	26.88	7.50	23.00	58.12				
	PVG	90	40.99	6.93	41.00	209.30	5,328.0	−6.502	0.000***	−0.36
	No PVG	223	34.89	7.11	35.00	135.89				
	PSMU	77	37.69	7.50	38.00	170.05	8,158.0	−1.397	0.162	
	No PSMU	237	36.28	7.49	36.00	153.42				
VGCH^2^	GTP	269	62.06	14.64	63.00	156.43	960.0	−5.910	0.000***	−0.34
	No GTP	25	39.60	14.65	36.00	51.40				
	PVG	87	74.78	9.35	77.00	233.59	2,211.0	−10.394	0.000***	−0.59
	No PVG	215	54.70	14.13	56.00	118.28				
	PSMU	78	56.41	15.38	56.50	128.56	6,947.0	−2.694	0.007*	−0.15
	No PSMU	224	61.53	15.67	63.00	159.49				
MUF^3^	GTP	271	26.03	7.33	25.00	147.88	3,220.0	−1.028	0.304	
	No GTP	27	26.59	3.74	27.00	165.74				
	PVG	89	25.40	7.03	24.00	144.79	8,881.0	−0.926	0.355	
	No PVG	214	26.20	7.04	25.00	155.00				
	PSMU	80	30.30	7.57	30.00	204.18	4,905.5	−6.052	0.000***	−0.35
	No PSMU	225	24.59	6.36	24.00	134.80				

*p < 0.05, **p < 0.01, ***p < 0.001.

*M, mean; SD, standard deviation; Me, median.*

*^1^Game engagement while playing, e.g., flow, losing track of time.*

*^2^Engagement in game-related activities and preference for playing.*

*^3^Media use questionnaire on frequency of use of social media platforms.*

*^*a*^Effect size = Cohen’s.*

*GTP, Game Transfer Phenomena; PSMU, problematic social media use; PVG, problematic video game playing; VGCH, Questionnaire on Video Game Consumption Habits; GEQ, Game Engagement Questionnaire; VGCH, Questionnaire on Video Game Consumption Habits; MUF, Media Use Frequency.*

#### Frequent Social Media Use

The Spearman rank-order correlations showed that only PSMU was positively correlated with MUF (Table 2).

Analyses conducted with the Mann–Whitney *U* test showed that when comparing the GTP vs. the No GTP group, the use of YouTube was statistically higher in the GTP group than in the No GTP group. Comparing the PVG groups showed that YouTube use was statistically higher in the PVG group than in the No PVG group. Comparing the PSMU groups showed that Facebook and Instagram use was statistically significantly higher in the PSMU group than in the No PSMU group. All the effect sizes were small, except for the medium effect size for the comparison in the PSMU group for Instagram use (see Table 6 for the full results).

**TABLE 6:** Mann–Whitney test of GTP, PVG, and PSMU on social media platforms.

Variables	Groups	*n*	*M*	*SD*	Me	Mean rank	*U*	*Z*	*p*	Effect size^a^
Facebook	GTP	295	4.54	2.381	5.00	162.16	3,788.0	−0.426	0.670	
	No GTP	27	4.37	2.420	5.00	154.30				
	PVG	95	4.40	2.410	5.00	163.14	10,938.5	−0.290	0.772	
	No PVG	235	4.62	2.341	5.00	166.45				
	PSMU	83	5.22	2.317	6.00	194.83	7,982.0	−3.150	0.002*	−0.17
	No PSMU	249	4.28	2.326	5.00	157.06				
LinkedIn	GTP	285	1.20	0.695	1.00	158.09	3,678.5	−1.263	0.207	
	No GTP	28	1.07	0.378	1.00	145.88				
	PVG	92	1.22	0.783	1.00	156.78	10,145.5	−0.773	0.439	
	No PVG	227	1.12	0.467	1.00	161.31				
	PSMU	83	1.11	0.350	1.00	158.41	9,662.0	−0.447	0.655	
	No PSMU	237	1.23	0.786	1.00	161.23				
Twitter	GTP	287	2.81	2.467	1.00	157.55	3,890.0	−0.301	0.764	
	No GTP	28	3.04	2.728	1.50	162.57				
	PVG	93	2.76	2.439	1.00	151.85	9,751.0	−1.219	0.223	
	No PVG	228	2.96	2.531	1.50	164.73				
	PSMU	83	3.45	2.773	2.00	181.69	8,242.5	−2.480	0.013	
	No PSMU	239	2.69	2.385	1.00	154.49				
Tumblr	GTP	287	1.75	1.581	1.00	159.17	3,682.0	−0.917	0.359	
	No GTP	28	1.46	1.071	1.00	146.00				
	PVG	93	1.67	1.510	1.00	160.11	10,519.0	−0.141	0.888	
	No PVG	228	1.76	1.603	1.00	161.36				
	PSMU	83	2.01	1.851	1.00	175.07	8,792.5	−1.965	0.049	
	No PSMU	239	1.63	1.443	1.00	156.79				
Instagram	GTP	294	2.66	5.23	6.00	158.86	3,339.5	−1.673	0.094	
	No GTP	28	6.11	2.378	6.00	189.23				
	PVG	95	4.94	2.710	6.00	148.00	9,500.0	−2.149	0.032	
	No PVG	235	5.72	2.572	6.00	172.57				
	PSMU	83	7.04	2.069	8.00	227.66	5,174.0	−6.877	0.000***	−0.38
	No PSMU	248	4.85	2.614	6.00	145.36				
YouTube	GTP	293	5.58	1.858	6.00	165.32	2,836.5	−2.783	0.005*	−0.16
	No GTP	28	4.61	2.006	5.00	115.80				
	PVG	94	5.94	1.771	6.00	194.86	8,144.5	−3.783	0.000***	−0.21
	No PVG	234	5.19	1.924	5.00	152.31				
	PSMU	82	5.45	2.240	6.00	164.46	10,083.0	−0.117	0.907	
	No PSMU	248	5.46	1.730	6.00	165.84				
Google Plus	GTP	288	2.23	2.059	1.00	156.11	3,344.0	−1.402	0.161	
	No GTP	27	3.00	2.557	1.00	178.15				
	PVG	94	2.04	1.894	1.00	150.20	9,654.0	−1.569	0.117	
	No PVG	227	2.49	2.234	1.00	165.47				
	PSMU	81	2.91	2.456	1.00	180.26	8,322.0	−2.353	0.019	
	No PSMU	242	2.18	1.981	1.00	155.89				
Pinterest	GTP	285	1.60	1.298	1.00	155.10	3,449.0	−1.497	0.134	
	No GTP	28	1.89	1.315	1.00	176.32				
	PVG	91	1.40	1.131	1.00	142.53	8,784.5	−2.681	0.007	
	No PVG	227	1.72	1.335	1.00	166.30				
	PSMU	83	1.87	1.606	1.00	173.40	8,682.0	−1.943	0.052	
	No PSMU	236	1.53	1.157	1.00	155.29				

*p < 0.05, **p < 0.01, ***p < 0.001.

*M, arithmetic mean; SD, standard deviation; Me, median.*

*^*a*^Effect size = Cohen’s.*

*GTP, Game Transfer Phenomena; PSMU, problematic social media use; PVG, problematic video game playing*.

### Predictors of Game Transfer Phenomena

In order to understand the interplay of personality traits, PVG and key gaming factors in those who experienced GTP, GTP as a binary outcome (i.e., GTP vs. no GTP), and the significant predictors for GTP were entered block-wise into a logistic regression using the Enter model. A parsimonious approach was taken for constructing the model that included a minimum number of predictor variables to understand if PVG, personality traits, and engagement increased the predictability of GTP. Those variables that were significantly correlated with GTP after the Bonferroni correction were entered into the model. Since this study has a particular interest to understand personality traits, personality traits that were higher for GTP than No GTP were also examined in the model.

The data were inspected for assumptions. The linearity of the continuous variables with respect to the logit of the dependent variable was assessed *via* the Box and Tidwell (1962) procedure. Only GE failed the assumption of linearity. Both VGCH and GE were excluded from the analyses. This decision was made for various reasons. Collinearity concerns were raised due to the large correlation between PSMU and VGCH (<7) and GE (<5) scores (see Table 2 for the correlation matrix), but the inflation rate in any case was not above 5. However, there were conceptual issues, as some items of the VGCH overlapped in conflicts derived from playing. Moreover, some of the items of GE overlap with losing control over playing, which is core to PVG. Therefore, no transformation was conducted trying to linearize the non-linear relationship of GE. None of the remaining variables had variance inflation rates (VIF) over 2, and the average VIF was 1.12, which indicates that the VIF was moderately correlated (Hilbe, 2016).

The predictors were entered into three blocks. Block 1 contained the personality traits according to the five-factor model that were higher in the GTP group and gender. This first block explained the 17% of variance of GTP (Nagelkerke’s *R*^2^ = 0.166); Model [χ^2^(3) = 22.423, *p* < 0.001]. Males were 5.2 times more likely than females to experience GTP. Every unit increase in neuroticism significantly increased the odds of GTP by 1.129 (12.9%). Openness was not a significant predictor of GTP.

In Block 2, fantasy proneness was additionally added, which increased the predicted power of the model when the BFI personality traits and gender were held constant. This block that contained all the variables in the model explained the 36% of the variance of GTP (Nagelkerke’s *R*^2^ = 0.361); Model [χ^2^(4) = 51.381, *p* < 0.001]. Males were then 13 times more likely than females to experience GTP, and every unit increase in fantasy proneness increased the odds of GTP by 1.489 (48.9%).

In Block 3, PVG was added while all the previous variables were held constant. The predicted power of the model increased even more and explained the 50% of the variance for GTP (Nagelkerke’s *R*^2^ = 0.496); Model [χ^2^(5) = 73.159, *p* < 0.001]. PVG emerged as the strongest predictor of GTP. Every unit increase in PVG significantly increased the odds of GTP by 2.391 (139.1%). Every unit increased in fantasy proneness significantly increased the odds of GTP by 1.300 (30.0%). Males were 4.3 times more likely than females to experience GTP. Neuroticism was no longer a predictor of GTP (see Table 7).

**TABLE 7:** Hierarchical binary logistic regression for GTP on dispositional factors.

	*B*	*(SE)*	Wald	df	Exp(B)
Gender (male)	1.654**	(0.481)	11.806	1	5.230
Openness	0.068	(0.037)	3.451	1	1.070
Neuroticism	0.122*	(0.043)	7.968	1	1.129
*Constant*	−3.779	(1.712)	4.873	1	0.023

*R*^2^ = 0.076 (Cox and Snell), 0.166 (Nagelkerke). Model [χ^2^(3) = 22.423, *p* < 0.001]					

Gender (male)	2.556***	(0.584)	19.183	1	12.883
Openness	−0.042	(0.049)	0.744	1	0.959
Neuroticism	0.093	(0.047)	3.994	1	1.097
CEQ	0.398***	(0.087)	21.137	1	1.489
*Constant*	−2.775	(1.870)	2.202	1	0.062

*R*^2^ = 0.165 (Cox and Snell), 0.361 (Nagelkerke). Model [χ^2^(4) = 51.381, *p* < 0.001]					

Gender (male)	1.451*	(0.625)	5.399	1	4.269
Openness	0.007	(0.055)	0.017	1	1.007
Neuroticism	0.072	(0.055)	1.675	1	1.074
CEQ	0.263*	(0.088)	8.916	1	1.300
PVG	0.872**	(0.236)	13.580	1	2.391
*Constant*	−4.858	(2.231)	4.741	1	0.008

*R*^2^ = 0.227 (Cox and Snell), 0.496 (Nagelkerke). Model [χ^2^(5) = 73.159, *p* < 0.001]					

*p < 0.05, **p < 0.01, ***p < 0.001.

*The predictors that were significantly associated with GTP (Game Transfer Phenomena) and personality traits that were higher in the GTP group than the No GTP group were entered into the logistic regression using the Enter method in a block-wise manner, beginning with the core personality traits of the Big Five personality traits and gender, followed by CEQ (Creative Experiences Questionnaire), as a measure of fantasy proneness, and finally PVG (problematic video game playing)*.

## Discussion

This study investigated the interrelationship and overlap between GTP with PVG and PSMU in a sample of video game players by focusing on dispositional factors and interactive media habits. The sample was relatively equally divided in terms of gender, and most participants were 25 years old or younger. Most played every day, and more than half played up to 2 h per day.

### The Prevalence and Relationship Between Game Transfer Phenomena, Problematic Video Game Playing, and Problematic Social Media Use

Similar to previous studies, the prevalence of GTP (having experienced at least one instance of GTP) was high (91.3%) (Ortiz de Gortari and Griffiths, 2016b; Dindar and Ortiz de Gortari, 2017). Most participants had experienced five or more different types of GTP. The most prevalent types of GTP were those that are also common among the general population (Ortiz de Gortari et al., 2016) such as earworms, visualizing or seeing images in the back of the eyelids as a sort of afterimage, and thinking about using video game elements in real-life contexts.

Previous research has shown inconsistencies in the prevalence of PVG and PSMU, the measurement of which substantially depends on the scales and thresholds used to confirm the conditions. Prior studies conducted with the scales that were chosen in the present study have reported a high prevalence of PVG (e.g., 8–23%) and PSMU (e.g., 12–22%) (Kuss et al., 2012; Lopez-Fernandez et al., 2014; Lin et al., 2017). Surprisingly, a remarkably high prevalence of PVG (28.5%) and PSMU (24.8%) was found in the present study even though conservative cutoff points were adopted. Hence, the findings should be interpreted with caution to avoid overestimation of problems associated with interactive technologies.

In line with a previous study (Ortiz de Gortari et al., 2016), PVG emerged as a predictor of GTP when taking into account personality traits. However, no associations were found with PSMU nor was PSMU associated with PVG. More specifically, not all who had experienced GTP were identified as having PVG or PSMU, but all of those in the PVG group had experienced GTP.

### Video Game and Social Media Habits

No significant associations were found in daily playing time, playing experience, and GTP, similar to a previous study (Dindar and Ortiz de Gortari, 2017). Interestingly, those who played 2–3 h or more per day and those who played every day were significantly more likely to have PVG. Playing less frequently, e.g., once a month, was more likely to be associated with PSMU.

That no significant association between gaming habits and GTP was found might suggest that individual factors (e.g., personality traits and cognitive profile) are even more relevant for GTP. However, one should be aware that the frequency of playing and session length have been found to predict severe levels of GTP (i.e., many types of GTP and a high frequency of occurrence) compared to low and moderate levels of GTP. Particularly, playing in a gaming session of 6 h or more was significantly associated with severe levels of GTP (Ortiz de Gortari et al., 2016).

In terms of social media use, Instagram, YouTube, and Facebook were the most frequently checked social/sharing media. The frequent use of social media was only significantly higher in those classified with PSMU. When taking a closer look at the platforms used by the sample, both those who experienced GTP and those with PVG had significantly higher scores on YouTube use compared to those with PSMU. This can be explained by the fact that YouTube is a popular outlet that is frequently used by players for watching and sharing videos on gameplay and news of upcoming games. High Facebook and Instagram scores were significantly associated with PSMU, which is understandable insofar as these platforms facilitate the interchange of text, images, and videos usually for socializing purposes.

### Video Game Engagement

Engagement, as a subjective experience while playing and engagement in game-related activities, and preference for playing were other factors investigated in the present study. Both types of engagement were significantly high for those who had experienced GTP and those who had PVG. However, engagement in game-related activities and preference for playing over other activities were significantly low in those with PSMU. High engagement in game-related activities and preference for playing over other activities by those who experienced GTP and those with PVG are not surprising.

Moreover, engagement, as measured along a unidimensional continuum gauging subjective phenomena such as flow, absorption, immersion, and presence, may make the game more gratifying and therefore more appealing to continue playing (Boyle et al., 2012). The detachment from reality and suspension of disbelief that occur with deep engagement while playing may make the individual more receptive to video game content and in-game experiences facilitating lingering effects such as those observed as GTP. For instance, playing for immersion (Ortiz de Gortari and Griffiths, 2015), immersion as a composed factor that includes losing track of time, forgetting what is happening around oneself, and mix-ups like looking outside the device screen to search for game elements have previously been found to be associated with GTP (Ortiz de Gortari, 2017).

### Gender and Personality Traits

The last research question in this study was concerned with dispositional factors that were assessed *via* demographics and personality traits. The only dispositional factors that have been investigated previously in the context of GTP are age and gender, and the majority of previous studies have found no association between gender and GTP (Ortiz de Gortari and Griffiths, 2016b; Dindar and Ortiz de Gortari, 2017; Ortiz de Gortari, 2017). However, in the present study, females were significantly less likely to experience GTP, which contradicts the findings of another study on a specific augmented reality game that showed that females were higher in most modalities of GTP (automatic mental processes, actions, and behaviors and visual and auditory sensory perceptions) (Sifonis, 2018). Further, in the present study, PVG was found to be prevalent among males, and PSMU was prevalent among females, which is consistent with previous studies (Andreassen, 2015; Andreassen et al., 2012, 2016, 2017).

Scores indicating being fantasy-prone were significantly higher for those who had experienced GTP. Fantasy-prone individuals are usually referred to as fantasizers, and they tend to spend a considerable amount of time fantasizing, recalling vivid childhood memories, and being susceptible to hallucinatory, out-of-body, paranormal, and intense religious experiences and hypnosis (Wilson and Barber, 1983). Furthermore, fantasy proneness has been associated with everyday slips and cognitive lapses (e.g., lapses in attention) (Merckelbach et al., 1991), and cognitive and sensory intrusions as those observed in GTP appear to be explained by failures in cognitive control (Ortiz de Gortari and Griffiths, 2019).

Finally, the logistic regression model for GTP revealed a deeper picture than the bivariate analyses in terms of the association between GTP, PVG, gender, and personality traits. The results of the model showed the relevance of dispositional factors (i.e., gender and personality traits) on GTP. When neuroticism and openness as part of the five core personality traits were examined, neuroticism emerged as a predictor of GTP, and males were more likely than females to experience GTP. Neuroticism has been associated with reports of vivid daydreaming and proneness to hallucinations (Larøi et al., 2005), which appear to be particularly relevant for GTP. Interestingly, when fantasy proneness came into the picture, it also emerged as a predictor of GTP, while being male was still a predictor, but this was not the case for neuroticism. Openness remained as not being a predictor of GTP.

Moreover, when PVG was included in the model, males remained more likely to experience GTP and only fantasy proneness was a predictor of GTP among the personality traits, and PVG became the strongest predictor of GTP. This may suggest that those with PVG who are fantasy-prone are the ones more susceptible to experience GTP. These results contrast the bivariate results that showed no significant association between PVG and fantasy proneness.

In summary, those who have experienced GTP and those with PVG were characterized by being male, having played video games for several years, and playing frequently. They were highly engaged in the game while playing (e.g., losing track of time), valued playing video games, and preferred playing video games over other activities. The differences between the GTP and the PVG groups were that while those in the GTP group were more likely to be fantasy-prone, those with PVG played 2–3 or more hours per day. The profiles of those experiencing GTP and PVG differed from those with PSMU, the latter of whom tended to be females and extroverts who seldom played video games, showed less preference for playing games, and frequently used social or sharing platform sites instead, such as Facebook and Instagram.

## Limitations

The empirical results reported herein should be considered in light of some limitations. First, regarding the sample selection, the generalizability of the study’s findings is limited, since the study used a convenience sample of university students whose participation was rewarded with course credits. This raises issues such as the potential for self-selection and social desirability biases. Furthermore, since the study focuses on video games, the analyses of PSMU are restricted to those participants who have PSMU and play games. This may limit comparisons with other studies where these criteria are not applied to those with PSMU. However, it is important to note that even in the segment of those with PSMU, the results in the current study are reported according to previous studies on PSMU.

Second, even though the scales for assessing PVG and PSMU have been widely used (e.g., Collins et al., 2012; Kuss et al., 2012; Tejeiro Salguero et al., 2012; Lopez-Fernandez et al., 2014; Andreassen et al., 2017; Bányai et al., 2017; Tejeiro Salguero and Vallecillo Gomez, 2020), there are limitations on the instruments chosen. For instance, the PVG was the first validated scale for assessing PVG and, therefore, has been influential in the research area. However, the scale is not entirely in line with the most recent debates and recommendations for tools that assess gaming disorder suggested by the DSM-5 and WHO. Although the PVG scale covers the core symptoms of gaming disorder delineated by WHO, such as having lower control over gaming and a priority for gaming despite the occurrence of negative consequences (World Health Organization (WHO), 2018), it also includes items not considered in the frame of addiction to assess the loss of control (e.g., “When I lose in a game, or I have not obtained the desired results, I need to play again to achieve my target”). This might explain the large number of participants classified with problematic interactive media consumption (i.e., either PVG or PSMU). Hence, the findings should be interpreted with caution and replicated using other scales to assess PVG.

Third, the criterion of having experienced at least one GTP was used to classify participants with GTP rather than considering the severity of GTP (e.g., several types of GTP and experiencing GTP very frequently), the latter of which might be more consistent with the extreme behaviors exhibited in problematic playing and gaming disorder. This decision probably contributes to the extensive overlap between GTP and PVG because all of those with PVG had experienced GTP. However, this decision was made for the following reasons: (i) it facilitated comparisons with previous studies at this early stage of the investigation of GTP, and (ii) only a few individuals tend to present severe levels of GTP (Ortiz de Gortari et al., 2016), and we did not have a large enough sample to represent this group in this study. In any case, it is important to notice that in the present study, more than half of the sample reported experiencing five or more types of GTP. Nevertheless, future studies should compare PVG with GTP in terms of varying degrees of severity.

Finally, the effect sizes of several comparisons suggest that the differences between the samples were small. The use of severity criteria for GTP and a scale with core factors for gaming disorder may yield larger effect sizes. Furthermore, to reduce the familywise error rate due to multiple comparisons, Bonferroni correction was applied. However, it is recommended that future studies examine all the significant variables and their relationship with personality traits, since this is the first time that these have been examined in the context of GTP. Examining the different modalities of GTP (sensory perceptions, cognitions, and behaviors) and personality would lead to an improved understanding of GTP, since the different forms of GTP may have different underpinnings.

## Conclusion

Despite the potential limitations considered above, it is notable that the results, to a large extent, are supported by findings in previous research into GTP and problematic interactive media use.

In terms of dispositional factors, this study showed that males are more likely to experience GTP, although this association between gender and GTP should be investigated even further, since different studies have shown inconsistent results.

Furthermore, the expected emergence of fantasy proneness as a predictor of GTP but the absence of openness should be investigated. Evidence has suggested that fantasizers tend to be open to the experiences (Merckelbach et al., 2001). However, some evidence has shown that fantasy proneness in adolescents is mainly related to neuroticism rather than openness (Sánchez-Bernardos and Avia, 2004). Future studies should also take a closer look at the five-factor model of personality, paying particular attention to neuroticism and openness and considering the different modalities of GTP (i.e., sensorial, automatic mental process and behaviors). Understanding the role of neuroticism in GTP is very important, since neuroticism denotes tendencies to respond with negative emotions to threat, frustration, or loss (Lahey, 2009), suggesting that individuals high in neuroticism traits may be more likely to experience GTP as aversive.

Overall, gender, personality traits, and PVG appear to predict the likelihood of experiencing GTP.

Beyond the contributions to the understanding of GTP, this study supports previous findings regarding personality factors, gender differences, and patterns of media use in individuals who play video games and those who have a preference for social networking. One of the most interesting findings was that those who play most per day were significantly more likely to have PVG, but not GTP.

This study can be viewed as an early step in differentiating GTP from problematic media use, in this case, PVG and PSMU. It showed the interplay between PVG and GTP, and that there is no relationship or similarity between PSMU and GTP, as denoted by the differences in personality traits, preference in media, and patterns of media use. Nevertheless, to better understand the relationship between GTP, problematic playing, and gaming disorder, the results should be tested again using a scale that is up-to-date and developed in accordance with the diagnostic criteria recommended for assessing gaming disorder. Moreover, different criteria for classifying GTP should be considered when comparing GTP and PVG groups. The severity levels of GTP should also be considered, which may facilitate the differentiation between GTP and PVG but most importantly because severe levels of GTP have been associated with distress and dysfunction and playing for sessions of 6 h or more.

As has been shown in this study, different factors influence GTP and PSMU. Thus, further research attempting to identify idiosyncratic characteristics influencing the occurrence of GTP could contribute to the theoretical differentiation between PSMU and problematic gaming.

Finally, future work can benefit from considering the role of GTP in gaming disorder, since intrusive thoughts, cognitive biases, and poor impulse control are pivotal in the initiation and maintenance of dysfunctional playing behaviors.

## Data Availability Statement

The raw data supporting the conclusions of this article will be made available by the authors, without undue reservation, to any qualified researcher.

## Ethics Statement

The studies involving human participants were reviewed and approved by Ethics Committee of MacEwan University. The patients/participants provided their written informed consent to participate in this study.

## Author Contributions

JG designed the study, obtained ethical approval, and supervised two students in mounting the survey online, cleaning the data, calculating subscale scores, and coding self-reports of dreams. Only parts of this study were used herein. AO conceptualized the background for the write-up, performed the statistical analysis, and wrote most of the article. Both authors contributed to the article and approved the submitted version.

## Conflict of Interest

The authors declare that the research was conducted in the absence of any commercial or financial relationships that could be construed as a potential conflict of interest.

## References

[B1] Ahn H. M. Chung H. J. Kim S. H. (2015). Altered brain reactivity to game cues after gaming experience. *Cyberpsychol. Behav. Soc. Netw.* 18 474–479. 10.1089/cyber.2015.0185 26252933

[B2] American Psychiatric Association (2013). *Diagnostic and Statistical Manual of Mental Disorders*, 5th Edn. Arlington, VA: American Psychiatric Publishing.

[B3] Andreassen C. S. (2015). Online social network site addiction: a comprehensive review. *Curr. Addict. Rep.* 2 175–184. 10.1007/s40429-015-0056-9

[B4] Andreassen C. S. Billieux J. Griffiths M. D. Kuss D. J. Demetrovics Z. Mazzoni E. (2016). The relationship between addictive use of social media and video games and symptoms of psychiatric disorders: a large-scale cross-sectional study. *Psychol. Addict. Behav.* 30 252–262. 10.1037/adb0000160 26999354

[B5] Andreassen C. S. Pallesen S. Griffiths M. D. (2017). The relationship between addictive use of social media, narcissism, and self-esteem: findings from a large national survey. *Addict. Behav.* 64 287–293. 10.1016/j.addbeh.2016.03.006 27072491

[B6] Andreassen C. S. Torsheim T. Brunborg G. S. Pallesen S. (2012). Development of a Facebook addiction scale. *Psychol. Rep.* 110 501–517. 10.2466/02.09.18.pr0.110.2.501-517 22662404

[B7] APA (2000). *Diagnostic and Statistical Manual of Mental Disorders – Text Revision*, Vol.IV. Washington, DC: American Psychiatric Association.

[B8] Armstrong R. A. (2014). When to use the Bonferroni correction. *Ophthalmic Physiol. Opt.* 34 502–508. 10.1111/opo.12131 24697967

[B9] Ballabio M. Griffiths M. D. Urbán R. Quartiroli A. Demetrovics Z. Király O. (2017). Do gaming motives mediate between psychiatric symptoms and problematic gaming? An empirical survey study. *Addict. Res. Theory* 25 397–408. 10.1080/16066359.2017.1305360

[B10] Bányai F. Zsila Á Király O. Maraz A. Elekes Z. Griffiths M. D. (2017). Problematic social media use: results from a large-scale nationally representative adolescent sample. *PLoS One* 12:e0169839. 10.1371/journal.pone.0169839 28068404 PMC5222338

[B11] Becerra F. L. (2012). Construcción y validación de un cuestionario sobre los hábitos de consumo de videojuegos en preadolescentes. *Rev. Electrón. Tecnol. Educ.* 40:a197. 10.21556/edutec.2012.40.361

[B12] Billieux J. Flayelle M. Rumpf H.-J. Stein D. J. (2019). High involvement versus pathological involvement in video games: a crucial distinction for ensuring the validity and utility of gaming disorder. *Curr. Addict. Rep.* 6 323–330. 10.1007/s40429-019-00259-x

[B13] Box G. E. Tidwell P. W. (1962). Transformation of the independent variables. *Technometrics* 4 531–550. 10.1080/00401706.1962.10490038

[B14] Boyle E. A. Connolly T. M. Hainey T. Boyle J. M. (2012). Engagement in digital entertainment games: a systematic review. *Comput. Hum. Behav.* 28 771–780. 10.1016/j.chb.2011.11.020

[B15] Brockmyer J. H. Fox C. M. Curtiss K. A. McBroom E. Burkhart K. M. Pidruzny J. N. (2009). The development of the game engagement questionnaire: a measure of engagement in video game-playing. *J. Exp. Soc. Psychol.* 45 624–634. 10.1016/j.jesp.2009.02.016

[B16] Buono F. D. Paul E. Sprong M. E. Smith E. C. Garakani A. Griffiths M. D. (2020). Gaming and gaming disorder: a mediation model gender, salience, age of gaming onset, and time spent gaming. *Cyberpsychol. Behav. Soc. Netw.* 23 647–651. 10.1089/cyber.2019.0445 32654509

[B17] Charlton J. P. Danforth I. D. (2007). Distinguishing addiction and high engagement in the context of online game playing. *Comput. Hum. Behav.* 23 1531–1548. 10.1016/j.chb.2005.07.002

[B18] Collin P. Rahilly K. Richardson I. Third A. (2011). *The Benefits of Social Networking Services.* Melbourne, VIC: Cooperative Research Centre for Young People, Technology and Wellbeing.

[B19] Collins E. Freeman J. Chamarro-Premuzic T. (2012). Personality traits associated with problematic and non-problematic massively multiplayer online role playing game use. *Pers. Individ. Dif.* 52 133–138. 10.1016/j.paid.2011.09.015

[B20] Cudo A. Wojtasiñski M. Tuṅnik P. Griffiths M. D. Zabielska-Mendyk E. (2020). Problematic Facebook use and problematic video gaming as mediators of relationship between impulsivity and life satisfaction among female and male gamers. *PLoS One* 15:e0237610. 10.1371/journal.pone.0237610 32810183 PMC7437455

[B21] Davis R. A. (2001). A cognitive-behavioral model of pathological Internet use. *Comput. Hum. Behav.* 17 187–195. 10.1016/s0747-5632(00)00041-8

[B22] Dindar M. Ortiz de Gortari A. B. (2017). Turkish validation of the game transfer phenomena scale (GTPS): measuring altered perceptions, automatic mental processes and actions and behaviours associated with playing video games. *Telemat. Inform.* 34 1802–1813. 10.1016/j.tele.2017.09.003

[B23] Dong Y. Peng C.-Y. J. (2013). Principled missing data methods for researchers. *SpringerPlus* 2:222. 10.1186/2193-1801-2-222 PMC3701793 23853744

[B24] Gackenbach J. Yu Y. Lee M.-N. Zhou Z. Yu G. (2016). Gaming, social media, and gender in Chinese and Canadian cultures. *Gend. Technol. Dev.* 20 243–278. 10.1177/0971852416660650

[B25] Gervasi A. M. La Marca L. Costanzo A. Pace U. Guglielmucci F. Schimmenti A. (2017). Personality and internet gaming disorder: a systematic review of recent literature. *Curr. Addict. Rep.* 4 293–307. 10.1007/s40429-017-0159-6

[B26] Griffiths M. D. Kuss D. J. Ortiz de Gortari A. B. (2017). Videogames as therapy: an updated selective review of the medical and psychological literature. *Int. J. Priv. Health Inf. Manag.* 5 71–96. 10.4018/ijphim.2017070105

[B27] Hall A. K. Chavarria E. Maneeratana V. Chaney B. H. Bernhardt J. M. (2012). Health benefits of digital videogames for older adults: a systematic review of the literature. *Games Health J.* 1 402–410. 10.1089/g4h.2012.0046 26192056

[B28] Hart G. M. Johnson B. Stamm B. Angers N. Robinson A. Lally T. (2009). Effects of video games on adolescents and adults. *Cyberpsychol. Behav.* 12 63–65. 10.1089/cpb.2008.0117 19006462

[B29] Hilbe J. M. (2016). *Practical Guide to Logistic Regression.* Boca Raton, FL: CRC Press.

[B30] John O. P. Srivastava S. (1999). The big five trait taxonomy: history, measurement, and theoretical perspectives. *Handb. Pers.* 2 102–138.

[B31] Kardefelt-Winther D. Heeren A. Schimmenti A. van Rooij A. Maurage P. Carras M. (2017). How can we conceptualize behavioural addiction without pathologizing common behaviours? *Addiction* 112 1709–1715. 10.1111/add.13763 28198052 PMC5557689

[B32] Krossbakken E. Pallesen S. Mentzoni R. A. King D. L. Molde H. Finserås T. R. (2018). A cross-lagged study of developmental trajectories of video game engagement, addiction, and mental health. *Front. Psychol.* 9:2239. 10.3389/fpsyg.2018.02239 30519203 PMC6258776

[B33] Kuss D. J. Griffiths M. D. (2012). Internet gaming addiction: a systematic review of empirical research. *Int. J. Ment. Health Addict.* 10 278–296. 10.1007/s11469-011-9318-5

[B34] Kuss D. J. Louws J. Wiers R. W. (2012). Online gaming addiction? Motives predict addictive play behavior in massively multiplayer online role-playing games. *Cyberpsychol. Behav. Soc. Netw.* 15 480–485. 10.1089/cyber.2012.0034 22974351

[B35] Kusse C. Shaffii-Le Bourdiec A. Schrouff J. Matarazzo L. Maquet P. (2012). Experience-dependent induction of hypnagogic images during daytime naps: a combined behavioural and EEG study. *J. Sleep Res.* 21 10–20. 10.1111/j.1365-2869.2011.00939.x 21848802

[B36] Laconi S. Tricard N. Chabrol H. (2015). Differences between specific and generalized problematic Internet uses according to gender, age, time spent online and psychopathological symptoms. *Comput. Hum. Behav.* 48 236–244. 10.1016/j.chb.2015.02.006

[B37] Lahey B. B. (2009). Public health significance of neuroticism. *Am. Psychol.* 64, 241–256. 10.1037/a0015309 19449983 PMC2792076

[B38] Larøi F. DeFruyt F. van Os J. Aleman A. Van der Linden M. (2005). Associations between hallucinations and personality structure in a non-clinical sample: comparison between young and elderly samples. *Pers. Individ. Dif.* 39 189–200. 10.1016/j.paid.2005.01.001 16778452

[B39] Lemmens J. S. Valkenburg P. M. Peter J. (2009). Development and validation of a game addiction scale for adolescents. *Media Psychol.* 12 77–95. 10.1080/15213260802669458

[B40] Lin C.-Y. Broström A. Nilsen P. Griffiths M. D. Pakpour A. H. (2017). Psychometric validation of the persian bergen social media addiction scale using classic test theory and rasch models. *J. Behav. Addict.* 6, 620–629. 10.1556/2006.6.2017.071 29130330 PMC6034942

[B41] Lopez-Fernandez O. (2018). Generalised versus specific internet use-related addiction problems: a mixed methods study on internet, gaming, and social networking behaviours. *Int. J. Environ. Res. Public Health* 15:2913. 10.3390/ijerph15122913 30572652 PMC6313434

[B42] Lopez-Fernandez O. Honrubia-Serrano M. L. Baguley T. Griffiths M. D. (2014). Pathological video game playing in Spanish and British adolescents: towards the exploration of Internet gaming disorder symptomatology. *Comput. Hum. Behav.* 41 304–312. 10.1016/j.chb.2014.10.011

[B43] Lopez-Fernandez O. Williams A. J. Kuss D. J. (2019). Measuring female gaming: gamer profile, predictors, prevalence, and characteristics from psychological and gender perspectives. *Front. Psychol.* 10:898. 10.3389/fpsyg.2019.00898 31105622 PMC6498967

[B44] Männikkö N. Ruotsalainen H. Miettunen J. Pontes H. M. Kääriäinen M. (2020). Problematic gaming behaviour and health-related outcomes: a systematic review and meta-analysis. *J. Health Psychol.* 25 67–81. 10.1177/1359105317740414 29192524

[B45] Mehroof M. Griffiths M. D. (2010). Online gaming addiction: the role of sensation seeking, self-control, neuroticism, aggression, state anxiety, and trait anxiety. *CyberPsychol. Behav. Soc. Netw.* 13 313–316. 10.1089/cyber.2009.0229 20557251

[B46] Merckelbach H. Horselenberg R. Muris P. (2001). The creative experiences questionnaire (CEQ): a brief self-report measure of fantasy proneness. *Pers. Individ. Dif.* 31 987–995. 10.1016/s0191-8869(00)00201-4

[B47] Merckelbach H. Muris P. Rassin E. (1999). Fantasy proneness and cognitive failures as correlates of dissociative experiences. *Pers. Individ. Dif.* 26, 961–967. 10.1016/S0191-8869(98)00193-7

[B48] Montag C. Bey K. Sha P. Li M. Chen Y. F. Liu W. Y. (2015). Is it meaningful to distinguish between generalized and specific Internet addiction? Evidence from a cross-cultural study from Germany, Sweden, Taiwan and China. *Asia Pac. Psychiatry* 7 20–26. 10.1111/appy.12122 24616402

[B49] Montag C. Flierl M. Markett S. Walter N. Jurkiewicz M. Reuter M. (2011). Internet addiction and personality in first-person-shooter video gamers. *J. Med. Psychol. Theor. Methods Appl.* 23:163. 10.1027/1864-1105/a000049

[B50] Montag C. Schivinski B. Sariyska R. Kannen C. Demetrovics Z. Pontes H. M. (2019). Psychopathological symptoms and gaming motives in disordered gaming—a psychometric comparison between the WHO and APA diagnostic frameworks. *J. Clin. Med.* 8:1691. 10.3390/jcm8101691 31618950 PMC6832511

[B51] Ortiz de Gortari A. B. (2017). Empirical study on game transfer phenomena in a location-based augmented reality game. *Telemat. Inform.* 35 382–396. 10.1016/j.tele.2017.12.015

[B52] Ortiz de Gortari A. B. (2018). “First insights into applying the game transfer phenomena framework for positive means,” in *Pervasive Computing Paradigms for Mental Health*, Vol. 253 eds Cipresso P. Serino S. Ostrovsky Y. Baker J. T. (Boston, MA: Springer).

[B53] Ortiz de Gortari A. B. (2019a). “Characteristics of game transfer phenomena in location-based augmented reality games,” in *Augmented Reality Games: Understanding the Pokémon GO Phenomenon*, ed. Geroimenko V. (Berlin: Springer International Publishing).

[B54] Ortiz de Gortari A. B. (2019b). “Game transfer phenomena: origin, development and contributions to the videogame research field,” in *Oxford Handbook of Cyberpsychology*, eds Attrill-Smith A. Fullwood C. Kuss D. Keep M. (Oxford: Oxford University Press), 10.1093/oxfordhb/9780198812746.001.0001

[B55] Ortiz de Gortari A. B. Aronsson K. Griffiths M. D. (2011). Game Transfer Phenomena in video game playing: a qualitative interview study. *International Journal of Cyber Behavior, Psychology and Learning* 1, 15–33.

[B56] Ortiz de Gortari A. B. Griffiths M. D. (2013). Altered Visual perception in game transfer phenomena: an empirical self-report study. *Int. J. Hum. Comput. Interact.* 30 95–105. 10.1080/10447318.2013.839900

[B57] Ortiz de Gortari A. B. Griffiths M. D. (2014). Automatic mental processes, automatic actions and behaviours in game transfer phenomena: an empirical self-report study using online forum data. *Int. J. Ment. Health Addict.* 12 432–452. 10.1007/s11469-014-9476-3

[B58] Ortiz de Gortari A. B. Griffiths M. D. (2015). Game transfer phenomena and its associated factors: an exploratory empirical online survey study. *Comput. Hum. Behav.* 51 195–202. 10.1016/j.chb.2015.04.060

[B59] Ortiz de Gortari A. B. Griffiths M. D. (2016a). Commentary: playing the computer game tetris prior to viewing traumatic film material and subsequent intrusive memories: examining proactive interference. *Front. Psychol.* 7:260. 10.3389/fpsyg.2016.00260 26941702 PMC4763333

[B60] Ortiz de Gortari A. B. Griffiths M. D. (2016b). Prevalence and characteristics of game transfer phenomena: a descriptive survey study. *Int. J. Hum. Comput. Interact.* 32 470–480. 10.1080/10447318.2016.1164430

[B61] Ortiz de Gortari A. B. Griffiths M. D. (2019). Letter to the Editor for ‘Current Addiction Reports’—Game Transfer Phenomena and Dissociation: a Reply to Guglielmucci et al. (2019). Current Addiction Reports.

[B62] Ortiz de Gortari A. B. Griffiths M. D. (2017). “Beyond the boundaries of the game: the interplay between in-game phenomena, structural characteristics of video games, and game transfer phenomena,” in *Boundaries of Self and Reality Online*, eds Gackenbach J. Bown J. (San Diego, CA: Academic Press), 97–121.

[B63] Ortiz de Gortari A. B. Oldfield B. Griffiths M. D. (2016). An empirical examination of factors associated with game transfer phenomena severity. *Comput. Hum. Behav.* 64 274–284. 10.1016/j.chb.2016.06.060

[B64] Ortiz de Gortari A. B. Pontes H. Griffiths M. D. (2015). The game transfer phenomena scale: an instrument for investigating the non-volitional effects of video game playing. *Cyberpsychol. Behav. Soc. Netw.* 18 588–594. 10.1089/cyber.2015.0221 26376231

[B100] Przybylski A. K. Weinstein N. Murayama K. (2017). Internet gaming disorder: investigating the clinical relevance of a new phenomenon. *Am. J. Psychiatry* 174, 230–236. 10.1176/appi.ajp.2016.16020224 27809571

[B65] Sánchez-Bernardos M. Avia M. (2004). Personality correlates of fantasy proneness among adolescents. *Pers. Individ. Dif.* 37 1069–1079. 10.1016/j.paid.2003.11.015

[B66] Sifonis C. M. (2018). Examining game transfer phenomena in the hybrid reality game, ingress. *Int. J. Hum. Comput. Interact.* 35 1–12.

[B67] Stavropoulos V. Gomez R. Mueller A. Yucel M. Griffiths M. (2020). User-avatar bond profiles: how do they associate with disordered gaming? *Addict. Behav.* 103:106245. 10.1016/j.addbeh.2019.106245 31891834

[B68] Stevens M. W. Dorstyn D. Delfabbro P. H. King D. L. (2020). Global prevalence of gaming disorder: A systematic review and meta-analysis. *Aust. N Z J. Psychiatry* 4867420962851. 10.1177/0004867420962851 33028074

[B69] Stickgold R. Malia A. Maguire D. Roddenberry D. O’Connor M. (2000). Replaying the game: hypnagogic images in normals and amnesics. *Science* 290 350–353. 10.1126/science.290.5490.350 11030656

[B71] Tejeiro Salguero R. A. Gómez-Vallecillo J. L. Pelegrina M. Wallace A. Emberley E. (2012). Risk factors associated with the abuse of video games in adolescents. *Psychology* 3:310. 10.4236/psych.2012.34044

[B72] Tejeiro Salguero R. A. Morán R. M. B. (2002). Measuring problem video game playing in adolescents. *Addiction* 97 1601–1606. 10.1046/j.1360-0443.2002.00218.x 12472644

[B73] Tejeiro Salguero R. A. Vallecillo Gomez J. L. (2020). El cuestionario problem video game playing (PVP): diez años de resultados de investigacion. *Rev. Estud. Campogibratareños* 41 147–156.

[B74] Wamsley E. J. Perry K. Djonlagic I. Reaven L. B. Stickgold R. (2010). Cognitive replay of visuomotor learning at sleep onset: temporal dynamics and relationship to task performance. *Sleep* 1 59–68. 10.1093/sleep/33.1.59 20120621 PMC2802248

[B75] Wilson S. C. Barber T. X. (1983). “The fantasy-prone personality: Implications for understanding imagery, hypnosis, and parapsychological phenomena,” in *Imagery: Current Theory Research, and Application*, ed. Sheikh A. A. (New York: Wiley), 10.1177/0004867420962851

[B76] World Health Organization (WHO) (2018). *Gaming Disorder.* Avaialble online at: http://www.who.int/features/qa/gaming-disorder/en/ (accessed January 10, 2020).

